# Is *Remusatia* (Araceae) Monophyletic? Evidence from Three Plastid Regions

**DOI:** 10.3390/ijms13010071

**Published:** 2011-12-22

**Authors:** Rong Li, Tingshuang Yi, Heng Li

**Affiliations:** 1 Key Laboratory of Biodiversity and Biogeography, Kunming Institute of Botany, Chinese Academy of Sciences, Kunming 650201, China; E-Mails: lirong@mail.kib.ac.cn (R.L.); tingshuangyi@mail.kib.ac.cn (T.Y.); 2 Germplasm Bank of Wild Species in Southwest China, Kunming Institute of Botany, Chinese Academy of Sciences, Kunming 650201, China

**Keywords:** Araceae, phylogeny, *Remusatia*, taxonomy

## Abstract

The genus *Remusatia* (Araceae) includes four species distributed in the tropical and subtropical Old World. The phylogeny of *Remusatia* was constructed using parsimony and Bayesian analyses of sequence data from three plastid regions (the *rbcL* gene, the *trnL-trnF* intergenic spacer, and the *rps16* intron). Phylogenetic analyses of the concatenated plastid data suggested that the monophyly of *Remusatia* was not supported because *R. hookeriana* did not form a clade with the other three species *R. vivipara*, *R. yunnanensis*, and *R. pumila*. Nevertheless, the topology of the analysis constraining *Remusatia* to monophyly was congruent with the topology of the unconstrained analysis. The results confirmed the inclusion of the previously separate genus *Gonatanthus* within *Remusatia* and disagreed with the current infrageneric classification of the genus.

## 1. Introduction

The herbaceous genus *Remusatia* Schott (Araceae) was established in 1832 and contains four species distributed in the tropical and subtropical Old World [1–4]. The species type *Remusatia vivipara* (Roxb.) Schott extends from Africa, Madagascar, southeastern Arabian Peninsula, and the Himalaya to southern China, and tropical Asia to Malesia, northern Australia, and the Pacific islands [2,4]. *Remusatia hookeriana* Schott and *R. pumila* (D. Don) H. Li and A. Hay are distributed in Nepal, Bhutan, Northeastern India, Southwestern China, and Northern Thailand [4]. *Remusatia yunnanensis* (H. Li and A. Hay) H. Li and A. Hay is endemic to Western Yunnan of China [4]. Species of *Remusatia* usually grow as epiphytes or lithophytes in a wide variety of habitats including forest, rocks and damp banks [1,2]. The tubers of *Remusatia vivipara* are used to treat mastitis, traumatic injuries, abscesses, and swellings [4].

*Remusatia* is characterized by a combination of characters including unbranched or branched stolons with numerous bulbils, peltate leaves, absent appendix at spadix, and entirely connate stamens [1,4]. Based on the position of the placenta, Li and Hay [5] divided *Remusatia* into two sections: sect. *Remusatia* and sect. *Gonatanthus* (Klotzsh) H. Li and A. Hay. Species of sect. *Remusatia* have parietal placenta and the inflorescence appearing before the leaf. This section includes *Remusatia vivipara* and *R. hookeriana*. Species of section *Gonatanthus* (*Remusatia pumila* and *R. yunnanensis*) have basal placenta and an inflorescence that appears with the leaf [5].

Historically, the group *Gonatanthus* was formally recognized as a separate genus with *Gonatanthus pumilus* (D. Don) Engler and Krause (=*Remusatia pumila*) as the type with basal placentation being the key difference [6–8]. However this difference was found to be inconsistent as placentation was observed to be either basal or parietal in species such as *Remusatia hookeriana* and *Gonatanthus yunnanensis* H. Li and A. Hay (=*R. yunnanensis*) [5]. As a result, the genus *Gonatanthus* was merged with *Remusatia* by Li and Hay [5]. Nevertheless, this classification needs to be further tested within a broader phylogenetic framework that includes many lineages from Araceae.

Previous molecular phylogenetic studies have only included a single species from *Remusatia* (*R. vivipara*). The most recent and comprehensive family-wide phylogenetic studies supported the placement of *Remusatia* in *Colocasia* clade or large *Pistia* clade based on coding (*rbcL* gene, *matK* gene) and non-coding (partial *trnK* intron, *trnL* intron, *trnL-F* spacer) plastid DNA sequences [9]. *Remusatia* has been suggested to be closely related to *Ariopsis* Nimmo, *Steudnera* K. Koch, and *Colocasia* Schott [9–11]. However, these previous studies focused on the relationships at tribal level and only sampled *Remusatia vivipara*. Species from section *Gonatanthus* have not been included. To gain a clearer understanding of the phylogenetic position of *Remusatia*, it is necessary to test its monophyly by including the remaining species.

In this study, we employ sequences of three coding or non-coding plastid regions (the *rbcL* gene, the *trnL-trnF* intergenic spacer, and the *rps16* intron) to construct the phylogeny of *Remusatia*, because these sequences have been shown to be useful for inferring relationships at the generic and specific levels of Araceae [9,11–17]. The objectives of this study are to (1) test the monophyly of *Remusatia* with all four species sampled, (2) confirm the combination of *Gonatanthus* with *Remusatia*, and (3) infer the phylogenetic relationships within the genus.

## 2. Results and Discussion

### 2.1. Results

We excluded the poly A or poly T, and the ambiguous alignment from the data sets (*rps16*, 25 bp between 195 and 219, 505 bp between 245 and 749, 29 bp between 791 and 819, and 6 bp between 1224 and 1229; *trnL-F*, 6 bp between 73 and 78, 4 bp between 126 and 129, 14 bp between 523 and 536, 211 bp between 696 and 906, 3 bp between 1096 and 1098, and 6 bp between 1102 and 1107). The aligned length, the numbers of variable and parsimony informative sites, and the best-fit model determined by Modeltest for each plastid region were given (Table 1). The variation among three regions is different and the *rps16* intron is the most variable region. The single-gene analysis using maximum parsimony and Bayesian methods demonstrated no significant incongruences for the phylogeny of *Remusatia* among the three regions (Figure S1–S3). Moreover, a quantitative approach using the incongruence length difference (ILD) test also indicated no conflict among the three data sets (*p* = 0.04). We thus concatenated the three plastid data set in our analysis.

**Figure 1:**
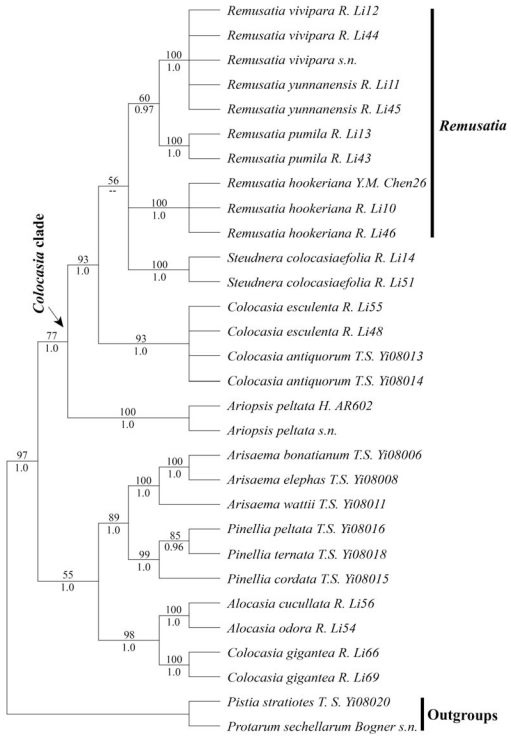
The parsimony strict consensus tree of *Remusatia* based on concatenated plastid data, with gaps treated as missing data (tree length = 412 steps, CI = 0.83, RI = 0.84, and RC = 0.70). Parsimony bootstrap values (PB) for maximum parsimony analysis in 500 replicates > 50% are shown above the branches and Bayesian posterior probabilities (PP) ≥ 0.95 are indicated below the branches. Double dash shows that the PP value was lower than 0.95.

The aligned length, the numbers of variable sites, and the numbers of parsimony informative sites for the concatenated plastid regions were given (Table 1). Treating gaps as missing data, the maximum parsimony analysis produced 3 most parsimonious trees (MPTs) of 412 steps, with a consistency index (CI) of 0.83, a CI excluding uninformative characters of 0.71, a retention index (RI) of 0.84, and a rescaled consistency index (RC) of 0.70. The Bayesian tree was nearly identical to the strict consensus tree of MPTs (Figure 1). Treating the gaps as new characters, the parsimony strict consensus tree also did not support the monophyly of *Remusatia* (Figure S4). Moreover, the resulting tree when gaps were treated as missing data is better resolved than the tree obtained with gaps as coded characters. For this reason, we discuss all results in this paper based on the analysis treating gaps as missing data (Figure 1).

The concatenated plastid data supported the monophyly of the *Colocasia* clade (PB = 77%, PP = 1.0). *Remusatia* was part of the *Colocasia* clade (Figure 1). The monophyly of *Remusatia* was not supported because *R. hookeriana* did not form a clade with other three species *R. vivipara*, *R. yunnanensis*, and *R. pumila* (see Figure 1). Excluding *Remusatia hookeriana*, the remaining *Remusatia* species formed a supported clade (PB = 60%, PP = 0.97) (Figure 1). Within this clade, *Remusatia pumila* is sister to the clade containing *R. vivipara* and *R. yunnanensis* (Figure 1).

Analyses constraining all *Remusatia* species into a clade generated MPTs that were the same tree length with the unconstrained MPTs. The SH test suggests topological congruence is well supported (*p* > 0.01) between constrained analysis and unconstrained analysis (Figure S5).

### 2.2. Discussion

#### 2.2.1. Is *Remusatia* Monophyletic?

With the current sampling, the monophyly of *Remusatia* is not supported. The species *R. hookeriana* does not group with the remainder of the *Remusatia* species sampled. However, the topology of the analysis constraining *Remusatia* to monophyly was congruent with the topology of unconstrained analysis (Figures 1 and S5). It is likely that the non-monophyly was due to the morphological and ploidy sampling bias, because our sampling for *R. hookeriana* remains poor (e.g., only three accessions having stolons branched and placentae parietal were sampled from *R. hookeriana*). *Remusatia hookeriana* is morphologically or cytologically variable in several characters including stolons simple or much branched, placentae parietal or basal, and chromosome numbers diploid or triploid [5,18–22]. These characters are shared with the remaining *Remusatia* species. It seems likely that *Remusatia hookeriana* may be more closely related to the common ancestor of the remaining *Remusatia* species. Thus, the monophyly of *Remusatia* needs to be further examined with additional *R. hookeriana* sampling covered the morphological or cytological diversity.

It is difficult to establish any morphological synapomorphies for the *Remusatia*, because the defining characters except the unbranched or branched stolons also occur in other genera of Araceae. These include peltate leaves, appendix absent at spadix, entirely connate stamens, and chromosome base number *x* = 14 [1,4]. At the family level, many of these morphological or cytological characters including leaf shape, spadix structure, male flowers morphology, and chromosome number have been shown to be relatively plastic [1,9].

In our phylogenetic tree, the genus *Remusatia* and the genus *Steudnera* form a weakly supported clade (PB = 56%, PP < 0.95) (Figure 1), which share the characters of the appendix absent at the spadix and numerous ovules. They differ in that *Steudnera* has stout rhizomes and absent stolons, whereas *Remusatia* possesses subglobose tubers and produces erect or spreading, unbranched or branched stolons from the axils of deciduous cataphylls [1,4]. The rarely flowering *Remusatia* species are spread by the generous formation of tubercles from stolons [23].

#### 2.2.2. Infrageneric Relationships Within *Remusatia*

Our phylogenetic analyses confirm the combination of previously separate genus *Gonatanthus* with *Remusatia*, because the *G. pumilus* (=*R. pumila*) (the type of *Gonatanthus*), *G. yunnanensis* (=*R. yunnanensis*), and *R. vivipara* (the type of *Remusatia*) formed a supported clade (Figure 1). Within this clade, *Remusatia pumila* is sister to the clade consisting of *R. vivipara* and *R. yunnanensis* (Figure 1). Morphologically, *Remusatia pumila* is distinguished from the other two species by not having a reflexed spathe with 2 constrictions (*vs.* reflexed spathe with only 1 constriction) [4,5]. *Remusatia vivipara* is phylogenetically close to *R. yunnanensis*, differing primarily in their stolon morphology and flowering time. *Remusatia vivipara* has erect, simple stolons and the inflorescence appearing before the leaf, whereas *R. yunnanensis* has creeping or pendulous, branched stolons and the inflorescence appearing together with the leaf [4,5].

In our study, the widely distributed *Remusatia vivipara* and narrowly endemic *R. yunnanensis* are represented by several accessions from Southern India and Yunnan in China. However, the monophyly of each species is not supported in the phylogenetic analyses. There may be several cryptic species in widespread *Remusatia vivipara* complex and in morphologically variable *R. yunnanensis* complex. Thus, a more thorough analysis (e.g., using genomic screening markers and additional taxon sampling) should be performed to confirm the status of *Remusatia vivipara* and *R. yunnanensis*.

Based on the placenta position, Li and Hay [5] split *Remusatia* into two sections: sect. *Remusatia* and sect. *Gonatanthus* (Klotzsh) H. Li and A. Hay. Our results do not support their infrageneric classification, because species with parietal or basal placentae do not form a clade. For example, *Remusatia yunnanensis* grouped with *R. vivipara* which has parietal placentae, rather than with *R. pumila* having basal placentae.

Below we provide a taxonomic key to the species of *Remusatia* to facilitate the identification of the four species [4]:

**Table:** 

1a. Spathe with two constrictions, one separating tube and limb, one separating limb into two parts	*R. pumila*
1b. Spathe with only one constriction separating tube and limb	
2a. Stonlons erect, simple, stout	*R. vivipara*
2b. Stonlons creeping or pendulous, simple or branched, slender	
3a. Limb of spathe semispreading to erect, not reflexed	*R. hookeriana*
3b. Limb of spathe initially erect, later reflexed	*R. yunnanensis*

## 3. Experimental Section

### 3.1. Taxon Sampling

Thirty accessions representing four species of *Remusatia* and 20 related taxa were included in this study (Table 2). Based on recent phylogenetic analyses of Araceae [9], the following genera were used as closely related taxa that represent major lineage within the large *Pistia* clade: *Ariopsis*, *Steudnera*, *Colocasia*, *Alocasia* (Schott) G. Don, *Arisaema* Martius, and *Pinellia* Tenore. Species of *Pistia* L. and *Protarum* Engl. were selected as outgroups because they have been shown to be outside the *Colocasia* clade within the large *Pistia* clade. The wide range of multiple taxa within the large *Pistia* clade was selected to further test the monophyly of *Remusatia* within a broader phylogenetic framework.

### 3.2. DNA Extractions, Amplification, and Sequencing

Total DNA was extracted from about 15 mg silica-gel dried leaf material using the DNeasy plant mini kits (QIAGEN, Mississauga, Ontario) following the manufacturer’s protocol or the modified CTAB extraction method [24].

Three coding or non-coding plastid regions (the *rbcL* gene, the *trnL-trnF* intergenic spacer, and the *rps16* intron) markers were employed in this study. The following primers were used for both amplification and sequencing: “1F” and “1460R” for the *rbcL* gene [25], “c” and “f” for the single *trnL-F* region or as two fragments with “c + d” and “e + f” [26]; when this region could not be amplified successful using primer “c”, we used instead primer “c2” [27], “F” and “R2” for the *rps16* intron [28]. Polymerase chain reaction (PCR) amplifications were performed in a 25 μL volume containing 1.5 mM MgCl_2_, 0.2 mM of each dNTP, 0.4 mM of each primer, 1 U of *Taq* polymerase (Bioline), and about 10–50 ng of DNA template under the following conditions: 3 min at 95 °C, followed by 37 cycles of 20 s at 94 °C, 30 s at 50 °C, and 40 s at 72 °C, and then a final 5 min extension at 72 °C.

The PCR products were purified using the polyethylene glycol (PEG) precipitation procedure following the protocol of Sambrook *et al.* [29]. Cycle sequencing was conducted using BigDye 3.1 reagents and carried out using the following profile: 35 cycles of 97 °C for 15 s, 50 °C for 5 s, and 60 °C for 4 min. The products of cycle-sequencing reactions were cleaned using the Sephadex columns (Amersham Pharmacia Biotech, Piscataway, New Jersey). The sequences were generated on an ABI prism 3730XL capillary sequencer (Applied Biosystems, Foster City, California). All sequences were newly generated in this study and have been deposited in GenBank (Table 2).

### 3.3. Sequence Alignment and Phylogenetic Analyses

The program Sequencher 4.5 (Gene Codes Corporation, Ann Arbor, Michigan) was used to evaluate chromatograms for base confirmation and to edit contiguous sequences. Sequences were initially aligned with ClustalX version 1.83 [30], followed by manual adjustments on Se-Al v2.0a11 [31].

To evaluate congruence of the three plastid (*rbcL*, *trnL-F*, *rps16*) data sets, we employed the partition homogeneity test or the incongruence length difference (ILD) test [32]. The partition homogeneity test was conducted with PAUP* version 4.0b10 [33] with 100 replicates, each with 100 random addition sequence replicates, tree bisection-reconnection (TBR) branch swapping, and keeping no more than 100 trees per random addition replicate. Following Cunningham [34], a significance level of *p* = 0.01 was adopted for this test.

Phylogenetic trees for each plastid region and the combined data set (concatenating the *rbcL*, *trnL-F*, and *rps16*) were constructed using maximum parsimony (MP) and Bayesian methods. The MP analyses was conducted using PAUP* version 4.0b10 [33]. All characters were weighted equally and gaps were treated as missing data and coded as binary characters for the concatenated plastid data set using the “simple gap coding” method [35]. The program GapCoder [36] was employed to score the insertions and deletions (indels). The most parsimonious trees were obtained with heuristic searches of 1000 replicates with random stepwise sequence addition, tree bisection-reconnection (TBR) branch swapping, collapse of zero-length branches, multiple tree option in effect, saving 100 trees from each random sequence addition. Parsimony bootstrap values (PB) for the clades [37] revealed in the most parsimonious trees (MPTs) were calculated with 500 bootstrap replicates. In each replicate, we performed 1000 random sequence addition replicates followed by tree bisection-reconnection (TBR) swapping, keeping no more than 10 trees per replicate. Tree statistics, including consistency index and the retention index, were calculated using PAUP*.

Modeltest 3.7 [38,39] was used to determine the optimal model of molecular evolution and gamma rate heterogeneity using the Akaike Information Criterion (AIC). Bayesian inference was implemented with MrBayes 3.1.2 [40] using a mixed model Bayesian analysis strategy. We assigned model parameters for each gene partition identified by AIC in Modeltest (Table 1). The Markov chain Monte Carlo (MCMC) algorithm was run for 10,000,000 generations with one cold and three heated chains, starting from random trees and sampling one out of every 1000 generations. Runs were repeated twice. The average standard deviation of split frequencies below 0.01 was examined to evaluate the convergence between the runs. The program Tracer 1.5 [41] was used to ensure that plots of the two analyses were converging on the same area and the log likelihoods had stabilized. The value of the effective sample size (ESS) for each statistic was above 200 after excluding the 25% burn-in. After discarding the trees saved prior to this point as burn-in, the remaining 7500 trees were used to construct majority-rule consensus trees using PAUP*. Nodes with posterior probabilities (PP) ≥ 0.95 in the consensus trees were considered statistically significant.

With *Remusatia* shown to be non-monophyletic in our initial analysis, we performed a constraint analysis using the concatenated plastid data set. With *Remusatia* constrained to be monophyletic, a parsimony analysis was performed with the heuristic search option using 1000 random sequence additions, TBR, and saving 100 trees from each random sequence addition. Shimodaira-Hasegawa (SH) test [42] was used to evaluate the topological congruence between gene trees produced by the likelihood method. The SH test was implemented in PAUP* with the best-fit model estimated using Modeltest 3.7 [38,39], RELL optimization, and 1000 bootstrap replicates to compare the difference between the RELL optimization and the computationally much more intensive full optimization. We compared the optimal trees (unconstrained) with constraint trees from the maximum likelihood analysis separately.

## 4. Conclusions

The present study constructed the first phylogeny of *Remusatia*. The monophyly of *Remusatia* was not supported by the concatenated plastid data (the *rbcL* gene, the *trnL-trnF* intergenic spacer, and the *rps16* intron). Phylogenetic analyses confirmed the combination of previous separate genus *Gonatanthus* with *Remusatia* and disagreed with the current infrageneric classification of the genus.

## Supplementary Materials



## Figures and Tables

**Table 1:** Characteristics of three plastid regions.

	Aligned length (bp)	Number of variable sites (%)	Number of parsimony-informative sites (%)	Model selected by AIC
*rbcL* gene	1221	80 (6.6%)	35 (2.9%)	HKY + I + G
*trnL-F* intergenic spacer	936	85 (9.1%)	45 (4.8%)	TVM + G
*rps16* intron	1014	160 (15.8%)	73 (7.2%)	TIM + G
Plastid concatenated	3171	325 (10.2%)	153 (4.8%)	

**Table 2:** Voucher information and GenBank accession numbers for *Remusatia* and related taxa used in this study. All collections are deposited at the Herbarium of Kunming Institute of Botany, Chinese Academy of Sciences (KUN).

Taxon	Voucher	Locality	GenBank Accession No.		

			*rbcL*	*trnL-F*	*rps16*
**Ingroups**					
*Alocasia cucullata* (Loureiro) G. Don	*R. Li 56*	China: Yunnan, Xishuangbanna	JQ237188	JQ237218	JQ237158
*Alocasia odora* (Roxburgh) K. Koch	*R. Li 54*	China: Yunnan, Xishuangbanna	JQ237190	JQ237220	JQ237160
*Ariopsis peltata* Nimmo	*H. AR 602*	S India	JQ237191	JQ237221	JQ237161
*Ariopsis peltata* Nimmo	*s.n.*	S India	JQ237192	JQ237222	JQ237162
*Arisaema bonatianum* Engler	*T.S. Yi 08006*	China: Yunnan, Gongshan	JQ237193	JQ237223	JQ237163
*Arisaema elephas* Buchet	*T.S. Yi 08008*	China: Yunnan, Baoshan	JQ237194	JQ237224	JQ237164
*Arisaema wattii* J. D. Hooker	*T.S. Yi 08011*	China: Yunnan, Gongshan	JQ237195	JQ237225	JQ237165
*Colocasia antiquorum* Schott	*T.S. Yi 08013*	China: Yunnan, Tengchong	JQ237199	JQ237229	JQ237169
*Colocasia antiquorum* Schott	*T.S. Yi 08014*	China: Yunnan, Yingjiang	JQ237200	JQ237230	JQ237170
*Colocasia esculenta* (L.) Schott	*R. Li 55*	China: Yunnan, Xishuangbanna, cultivated	JQ237189	JQ237219	JQ237159
*Colocasia esculenta* (L.) Schott	*R. Li 48*	China: Yunnan, Lvchun, cultivated	JQ237196	JQ237226	JQ237166
*Colocasia gigantea* (Blume) J. D. Hooker	*R. Li 66*	China: Yunnan, Lvchun	JQ237197	JQ237227	JQ237167
*Colocasia gigantea* (Blume) J. D. Hooker	*R. Li 69*	China: Yunnan, Jinping	JQ237198	JQ237228	JQ237168
*Pinellia cordata* N. E. Brown	*T.S. Yi 08015*	China: Fujian, Wuyishan	JQ237201	JQ237231	JQ237171
*Pinellia peltata* C. Pei	*T.S. Yi 08016*	China: Zhejiang, Wenzhou	JQ237202	JQ237232	JQ237172
**Ingroups**					
*Pinellia ternata* (Thunb.) Tenore ex Breitenbach	*T.S. Yi 08018*	China: Yunnan, Kunming	JQ237203	JQ237233	JQ237173
*Remusatia hookeriana* Schott	*Y.M. Chen 26*	China: Yunnan, Wuding	JQ237206	JQ237236	JQ237176
*Remusatia hookeriana* Schott	*R. Li 10*	China: Yunnan, Jinping	JQ237207	JQ237237	JQ237177
*Remusatia hookeriana* Schott	*R. Li 46*	China: Yunnan, Lvchun	JQ237208	JQ237238	JQ237178
*Remusatia pumila* (D. Don) H. Li & A. Hay	*R. Li 13*	China: Yunnan, Pingbian	JQ237209	JQ237239	JQ237179
*Remusatia pumila* (D. Don) H. Li & A. Hay	*R. Li 43*	China: Yunnan, Lvchun	JQ237210	JQ237240	JQ237180
*Remusatia vivipara* (Roxb.) Schott	*R. Li 12*	China: Yunnan, Jinping	JQ237211	JQ237241	JQ237181
*Remusatia vivipara* (Roxb.) Schott	*R. Li 44*	China: Yunnan, Lvchun	JQ237212	JQ237242	JQ237182
*Remusatia vivipara* (Roxb.) Schott	*s.n.*	S India	JQ237213	JQ237243	JQ237183
*Remusatia yunnanensis* (H. Li & A. Hay) H. Li & A. Hay	*R. Li 11*	China: Yunnan, Yingjiang	JQ237214	JQ237244	JQ237184
*Remusatia yunnanensis* (H. Li & A. Hay) H. Li & A. Hay	*R. Li 45*	China: Yunnan, Yingjiang	JQ237215	JQ237245	JQ237185
*Steudnera colocasiifolia* K. Koch	*R. Li 14*	China: Yunnan, Puer	JQ237216	JQ237246	JQ237186
*Steudnera colocasiifolia* K. Koch	*R. Li 51*	China: Yunnan, Xishuangbanna	JQ237217	JQ237247	JQ237187
**Outgroups**					
*Pistia stratiotes* L.	*T.S. Yi 08020*	China: Yunnan, Kunming	JQ237204	JQ237234	JQ237174
*Protarum sechellarum* Engl.	*Bogner s.n.*	Germany: Munich Botanical Garden, cultivated	JQ237205	JQ237235	JQ237175
