# Supplementary material for: Is Remusatia (Araceae) Monophyletic? Evidence from Three Plastid Regions
Source: Int J Mol Sci. 2011 Dec 22;13(1):71–83. doi: 10.3390/ijms13010071 (PMC3269673; doi:10.3390/ijms13010071)
Supplement: Supplementary file 1 [file ijms-13-00071-s001.pdf]

Supplementary Materials

Figure S1. The parsimony strict consensus tree of *Remusatia* based on the *rbcL* gene, with gaps treated as missing data (tree length = 106 steps, CI = 0.81, RI = 0.83, and RC = 0.68). Parsimony bootstrap values (PB) for maximum parsimony analysis in 500 replicates > 50% are shown above the branches and Bayesian posterior probabilities (PP) ≥ 0.95 are indicated below the branches. Dashed lines indicate the branches that are not supported by both maximum parsimony and Bayesian analysis.

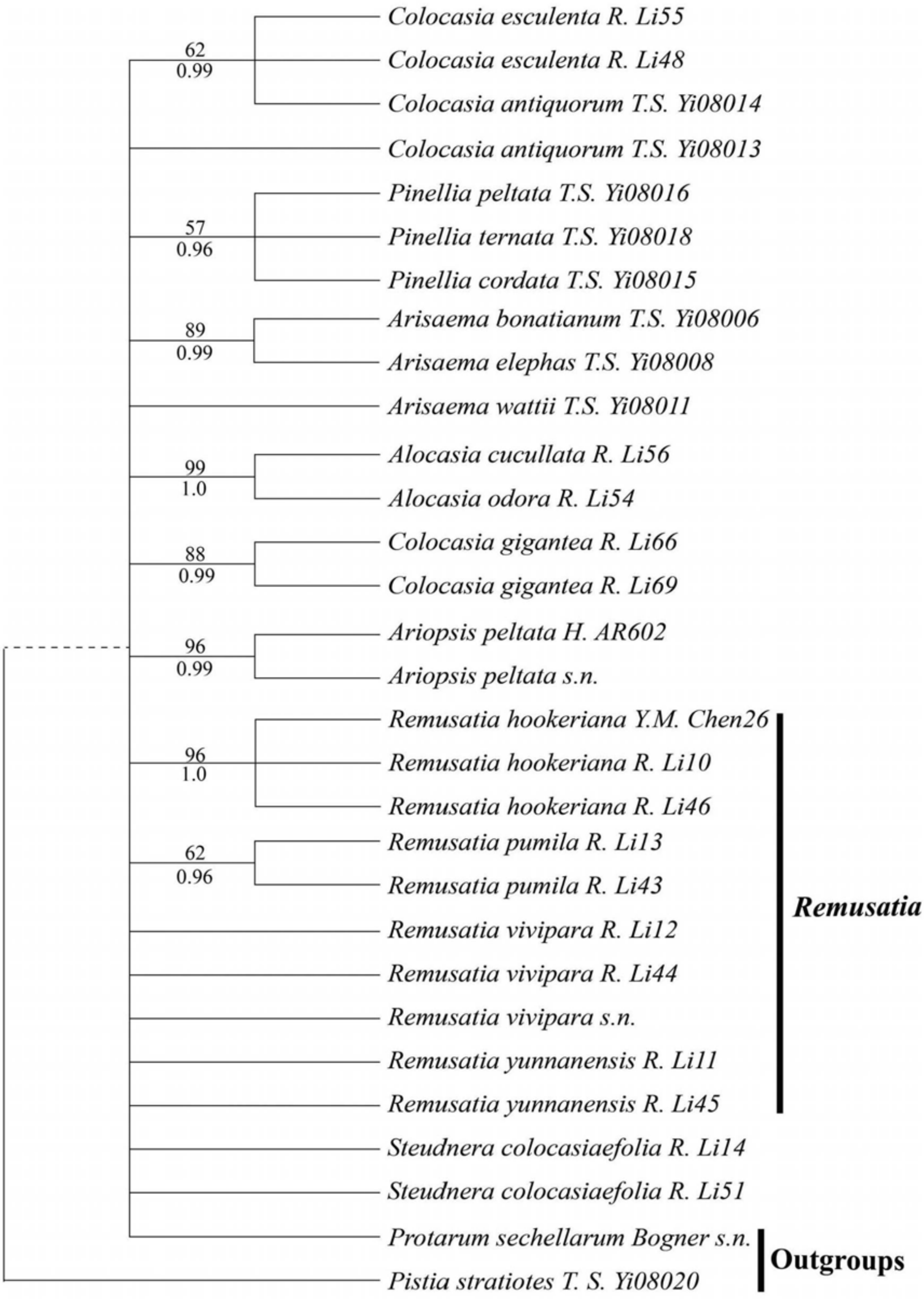

Figure S2. The parsimony strict consensus tree of *Remusatia* based on the *trnL-F* intergenic spacer, with gaps treated as missing data (tree length = 98 steps, CI = 0.90, RI = 0.91, and RC = 0.82). Parsimony bootstrap values (PB) for maximum parsimony analysis in 500 replicates > 50% are shown above the branches and Bayesian posterior probabilities (PP) ≥ 0.95 are indicated below the branches. Double dash shows that the PP value was lower than 0.95. Dashed lines indicate the branches that are not supported by both maximum parsimony and Bayesian analysis.

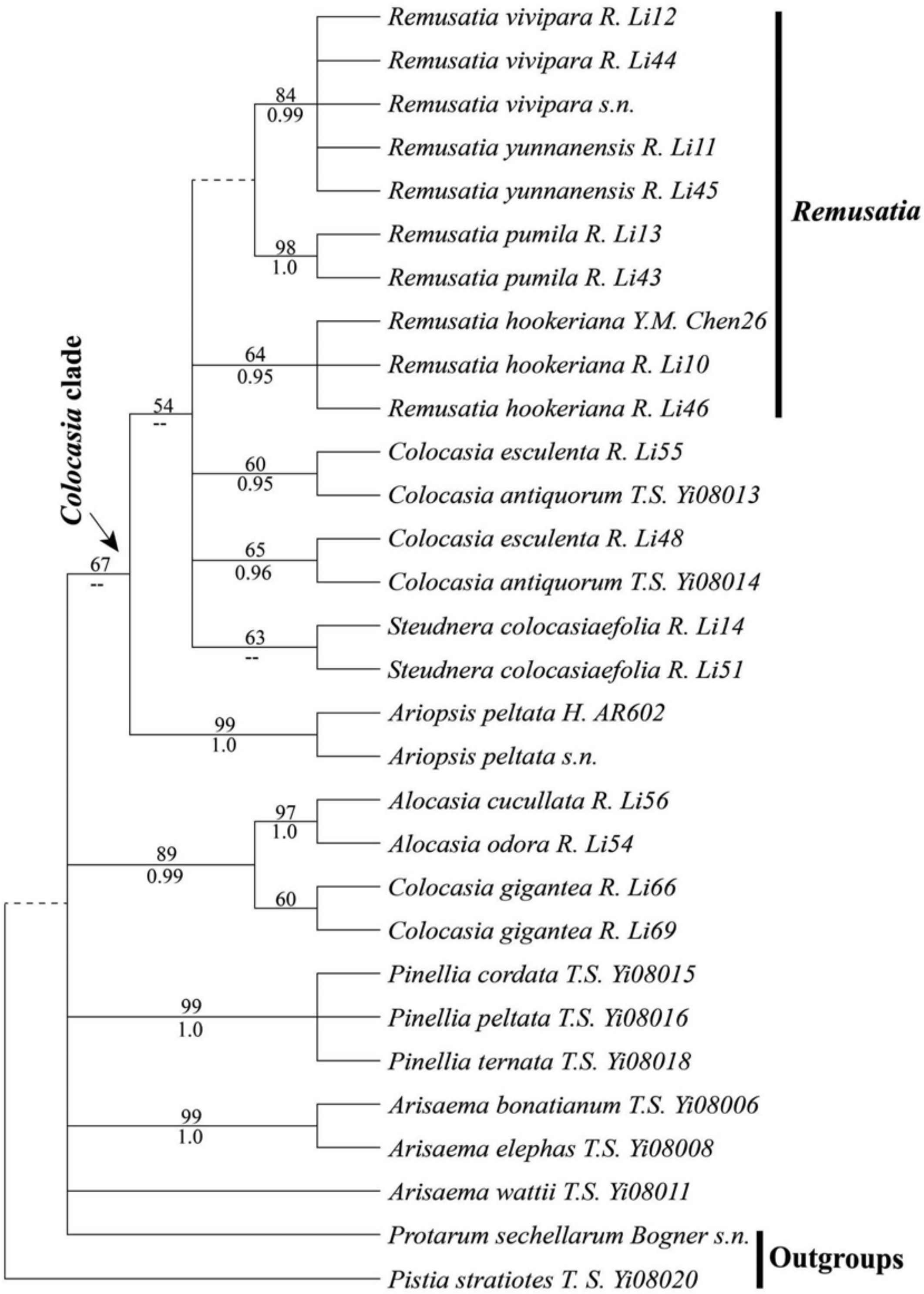

Figure S3. The parsimony strict consensus tree of *Remusatia* based on the *rps16* intron, with gaps treated as missing data (tree length = 200 steps, CI = 0.85, RI = 0.84, and RC = 0.71). Parsimony bootstrap values (PB) for maximum parsimony analysis in 500 replicates > 50% are shown above the branches and Bayesian posterior probabilities (PP) ≥ 0.95 are indicated below the branches. Double dash shows that the PP value was lower than 0.95.

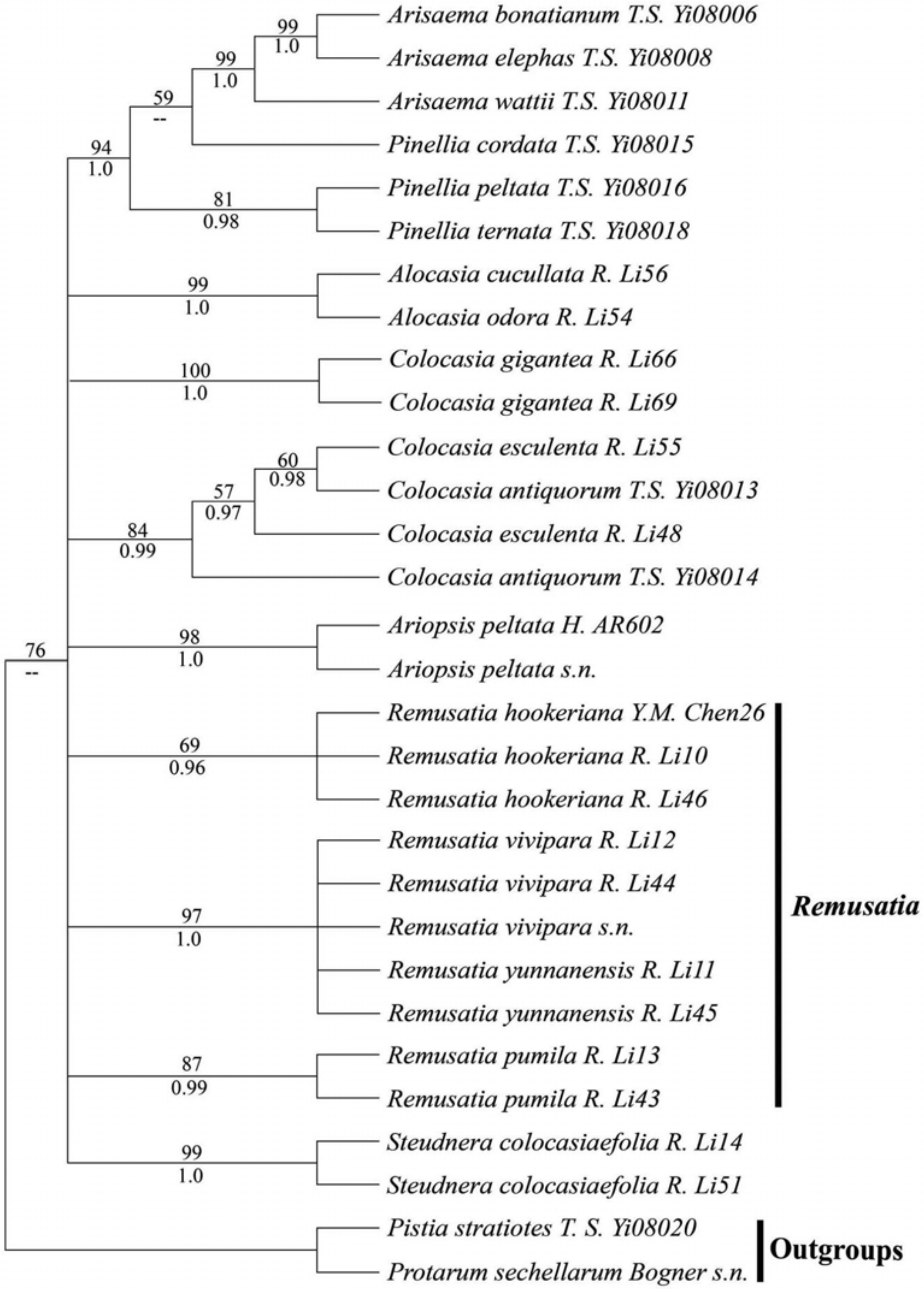

Figure S4. The parsimony strict consensus tree of *Remusatia* based on concatenated plastid data, with gaps treated as new characters (tree length = 484 steps, CI = 0.82, RI = 0.84, and RC = 0.69). Parsimony bootstrap values (PB) for maximum parsimony analysis in 500 replicates > 50% are shown above the branches and Bayesian posterior probabilities (PP) ≥ 0.95 are indicated below the branches. Double dash shows that the PB value was lower than 50% or the PP value was lower than 0.95. Dashed lines indicate the branches that are not supported by both maximum parsimony and Bayesian analysis.

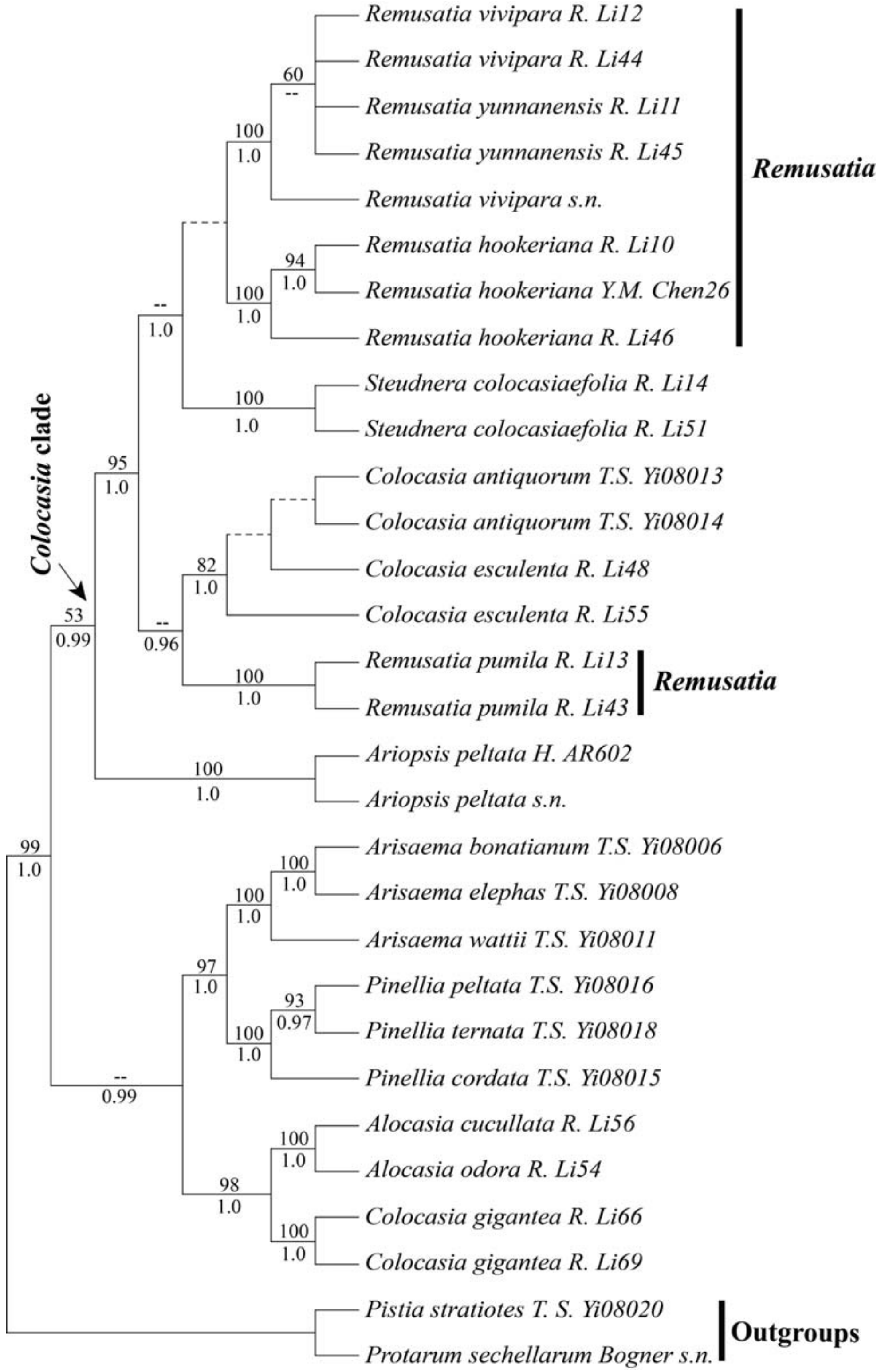

Figure S5. The parsimony strict consensus tree of constraining all *Remusatia* species into a clade based on concatenated plastid data, with gaps treated as missing data (tree length = 412 steps, CI = 0.83, RI = 0.84, and RC = 0.70). Parsimony bootstrap values (PB) for maximum parsimony analysis in 500 replicates > 50% are shown above the branches.

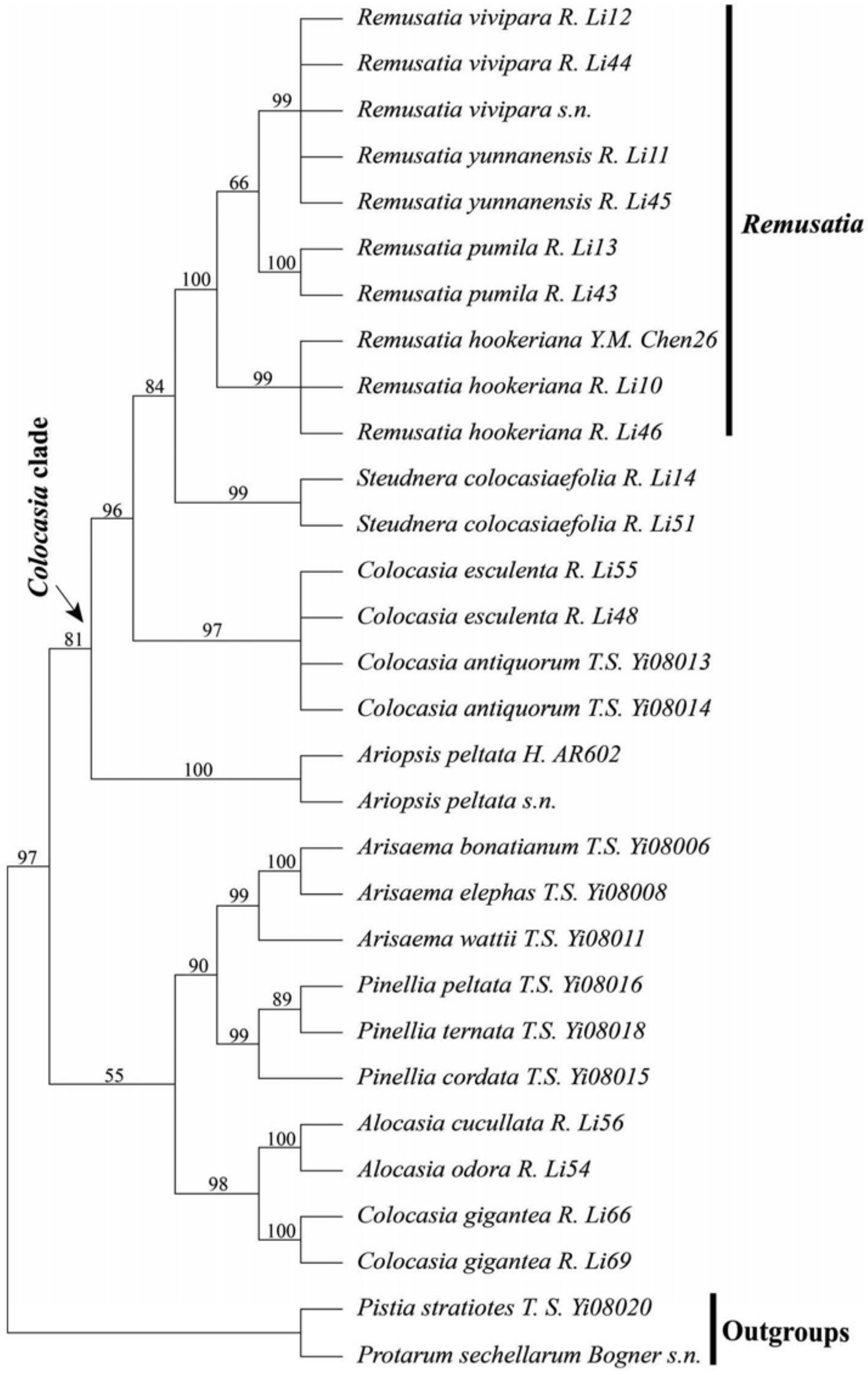

© 2012 by the authors; licensee MDPI, Basel, Switzerland. This article is an open access article distributed under the terms and conditions of the Creative Commons Attribution license (http://creativecommons.org/licenses/by/3.0/).
